# An *in vitro* assessment of glass ionomer cement shear bond-strength to demineralized dentin in primary teeth treated with silver diamine fluoride and potassium iodide

**DOI:** 10.6026/9732063002001046

**Published:** 2024-09-30

**Authors:** Jayatri Mondal, Rachana Bahuguna, Adnan Ahmed, Patatri Mitra, Prajakta Bhaskar Mahajan, Ananya Mishra

**Affiliations:** 1 Department of Pedodontics and Preventive Dentistry, Maitri College of Dentistry and Research Centre, Anjora, Durg, Chhattisgarh, India-491001; 2 Department of Conservative Dentistry and Endodontics, Maitri College of Dentistry and Research Centre, Anjora, Durg, Chhattisgarh, India-491001

**Keywords:** Silver diamine fluoride, potassium iodide, deciduous teeth, type IX-GIC, shear bond strength

## Abstract

Comprehensive caries therapy has relied on eliminating contaminated tooth structure and replacing it with restorative materials in pediatric dentistry. SDF with GIC can be administered together to stop the caries in cavitated lesions. Therefore, it is of interest to assess the shear bond strength of glass ionomer cement (type IX) to demineralized dentin in primary teeth treated with SDF and KI. The occlusal edges of sixty non-carious primary molar teeth were sliced until the yellow dentin was discernible. Each sample was embedded in an acrylic block with its occlusal surface facing upward after being submerged in a demineralizing solution for 7 days. Three groups were created from the samples: Group 1: The untreated group (control group) to which GIC type IX was placed directly; Group 2: Samples received immediate application of GIC type IX after getting treated with silver diamine fluoride (Experimental group) and Group 3: Silver diamine fluoride followed by potassium iodide followed by GIC type IX application (Experimental group). A Universal Testing Machine was subsequently utilized to perform a shear bond test on the specimens. Data shows that previously treated cement with SDF+KI increases the shear bond test without negatively impacted the bond strength GIC to dentin.

## Background:

The most prevalent chronic childhood disease, dental caries, is becoming more widespread among children between the ages of two and five globally; a population that need immediate attention [1]. Early childhood caries is one of the most often addressed subjects in dental literature, particularly in developing nations. Owing to its chronic nature, the most prevalent childhood illness has negative effect on children's quality of life in relation to oral health by affecting their food intake, sleep patterns, and general well-being. Over time, it has a negative impact on the parents as well. Strategies created to lessen the chronic nature of ECC have transformed into the use of minimally invasive techniques capable of treating the illness rather than just preventing it [2]. Throughout past 100 years, the management of dental caries has undergone a radical transformation. The classic "drill and fill" method utilised in this treatment was based on G.V. Black cavity design with "extension for prevention" concepts [3]. Japan was the first country to legalize the use of diamine silver fluoride (Ag (NH3) 2F) (SDF) for medicinal purposes in 1960s [4]. SDF is a water-based remedy that possesses the remineralizing characteristics of fluoride (F) and the antimicrobial abilities of silver (Ag). The Food and Drug Administration of the United States (FDA) approved the first SDF agent for commercial use as a Class-II dental product to treat tooth hypersensitivity in August 2014. American Dental Association, or "ADA", states that SDF is a commendatory material for treating dentin-sensitive areas in particular dental circumstances, like children undergoing surgery, special needs dentistry. It is also beneficial for preventing and/or managing caries disease [5]. One of the primary drawbacks of SDF treatment is the discoloration of dental tissues and restoration products caused by leftover silver ions. It was discovered that using potassium iodide (KI) can decrease discoloration caused by remaining silver ions by forming a washable creamy yellowish product called silver iodide [6]. The other alternate option is to use direct aesthetic restorations, such as GIC restorations, after applying SDF in order to conceal the black staining generated by the SDF while also improving aesthetics and chewing ability. Combining these two approaches, silver-modified A-traumatic restorative treatment (SMART) has been demonstrated to be effective in preventing dentin caries in children's primary teeth [7-8]. The acceptability of SDF with GIC restorations has been demonstrated by numerous research, nevertheless, little is known regarding the effect of discoloration-masking additives on bond strength amongst GIC and caries-affected dentine (CAD) that has previously received SDF, KI treatment.

The bond durability of a GIC restoration to demineralized dentin was not significantly affected by SDF treatment of artificial carious dentin in permanent teeth, according to Zhao *et al.* [8] and Ng *et al.* [9] Therefore, it is of interest to explore the implications of SDF and KI pre-treatment on the shear bond strength (SBS) of GIC (type IX) to demineralized dentin.

## Methods:

## Selection of teeth:

This research study was undertaken in the Department of Pedodontics and Preventive Dentistry of Maitri College of Dentistry and Research Centre, Anjora, Durg, Chhattisgarh. 60 primary molar was selected using an alpha (α) level of 0.05. Beta (β) level of 0.2 *i.e.*, Power = 80%. For each category, the minimum projected sample size is twenty samples. The sample size computation was done with G*Power 3.1.9.4.

## Inclusion criteria:

[1] Sound primary molar teeth which have exfoliated naturally

[2] Indicated for extraction due to over-retention

[3] For orthodontic purposes

## Exclusion criteria:

[1] Primary teeth previously infected with caries

[2] Restored teeth

[3] Fractured teeth

## Study design:

## Specimen Preparation:

To create a homogenous dentin surface, occlusal enamel was subsequent to that, cut until yellow dentin was visibleusing a slow-speed diamond disk and water as coolant. Next, using silicon carbide paper (600grit) and a water-cooled lathe, the dentin surfaces were polished and abraded to reveal a flat dentin surface. To ensure that no enamel remained, the uncovered tooth surfaces were examined under a light microscope. To leave only the exposed flat dentin surface, the specimens were coated with nail varnish (Revlon, NY, USA). Using a pH cycling technique, artificial dentine carious lesions were created by immersing the specimens for 7 days in demineralizing solution (pH 4.4, 50 mM acetate, 2.2 mM KH2 PO4, 2.2 mM CaCl2). Next, with the occlusal surface facing upward, each sample was embedded in an acrylic block of 1.5 cm by 1.5 cm by 2.5 cm.

## Grouping of teeth:

The samples were then divided randomly into three groups depending on pre-treatment with various solutions (n = 20 per group):

Group 1 (Control group)- Untreated group to which GIC type IX was applied directly to the demineralized dentinal surface by mixing powder and liquid were in a 3:1 ratio.

Group 2 (Experimental group) (SDF)- Glass ionomer cement was used to restore the demineralized edges of all the samples in this group after they had been coated with a 38% SDF (e-SDF) remedy using a brush tip for one minute. This was immediately followed by a 30-second rinsing with distilled water.

Group 3 (Experimental group) (SDF-KI)- After applying 38% SDF solution to the demineralized surfaces, a separate micro brush was used to apply the KI solution (Lugol's solution 10 wt%, Nice Chemicals, Kochi, India) until the creamy white precipitates became apparent. After giving the surfaces, a thorough 30 second cleaning with distilled water, they were set to air dry.

All the freshly demineralized dentine samples were bonded with GICs using a typical plastic mold that was 4 mm in diameter and 3 mm in height when it was placed into the dentin surface. Blade No. 15 (Sterilance Medical Suzhou Inc.) was used to remove the plastic mold that had encased the glass ionomer cylindrical buttons after bonding. To enable the complete setting of GICs, every sample was stored for a full day at 37°C and 100% humidity. After that, the specimens underwent 500 cycles of thermocycling (Make: LG Model: 051SA) at 5° to 55°C for 60 seconds. Mahavir, India) had a temperature of 5°C and dwell time of 30 seconds.

## Shear bond strength testing:

The Universal Testing Machine (Model: UNITEST 10, Computerized, Software-Based, ACME Engineers, India) has a system accuracy of ± 1%. After the specimens were exposed for a full day, the shear bond test was conducted using a flat-edge loading head. The loading head moved when a shear force was applied diagonally to the GIC spherical press at an ongoing rate of 1 mm per minute, leaving a gap of 1 mm between it and the dentin surface. The force in Newtons that was required to debone GICs was noted. Mega-Pascals (MPa) were used to express the bond strength as the result of multiplying the load at collapse by the connected area expressed in square millimeters.

Stress = Failure load (N)/surface area (mm^2^)

## Results:

## Statistical analysis:

Standard deviation ± mean was used to express the data. To determine the significance of the difference, an Anova test was employed, as the study contains more than two groups. P Value < 0.05 was considered to be statistically significant at 95% confidence interval. Additional statistical computations were performed employing MS Excel® (Microsoft Corp., New Mexico, USA) and SPSS®23 (IBM Corp., NY, USA). Table 1 displays the standard shear bond strengths of GICs to dentine. The mean is the highest with Group 3 (1.93±0.34), followed by Group 1 (1.49±0.38) and the least value of SBS has been recorded with Group 2 (1.09±0.32). Table 2 represent that pairwise comparison between shear bond strength of Group 1 GIC was done with Group 2 SDF + GIC and Group 3, the difference in mean was 0.40 & -0.43 respectively, between Group 2 and Group 3 was done the difference in mean was -0.83.The results indicate a substantial difference between the evaluations of all the groups.

## Discussion:

The treatment of carious lesions is approached less invasively in the principles of restorative dentistry. Since only the moistened, softened dentin is heavily contaminated by bacteria, as long as a biological seal is established and maintained, removing this layer alone will guarantee the caries-arrestment process. In order to maximize the tooth's ability to repair, it is advised to conserve both healthy dental tissues and tissues that have the capacity to re-mineralize [10]. The 1980s saw the development of atraumatic restorative treatment (ART), a minimally invasive surgical method for treating cavitated dentin carious lesions. Single-surface ART restorations are recommended for use in clinical practice among permanent and primary teeth because of their good retention and survival rate. In order toenhance the appearance and functionality of the teeth with the goal to restore the tooth's shape and hide the black stain, dentin lesions caused by caries that have been handled by SDF can be repaired using the ART procedure. It is crucial to ascertain whether dentin's surface SDF treatment affects the dentin's bonding strength to GIC [11]. Early in the 1970s, the Ministry of Health and Welfare in Japan's Central Pharmaceutical Council approved silver diamine fluoride (Ag (NH3)2F)) as a therapeutic agent. Up until the age of three, children with intermediate to high caries activity were advised to use SDF through the "Brazilian assistance program" [12].

Current research demonstrates that silver ions interfere with the formation of biofilms by interfering with the bacterial manufacture of cellular polysaccharides. Furthermore, since glucan makes up the majority of the biofilm, silver also interferes with the glucosyl transferase enzyme, which is responsible for glucan synthesis. By replacing the apatite crystals in the acid, the fluoride ions in the SDF prevent demineralization and encourage remineralization. A squamous layer of silver-protein conjugates generated on a degraded surface when silver diamine fluoride is put down, strengthening the surface's resistance to enzymatic digestion and acid breakdown. Silver chloride and metallic silver have been detected, and hydroxyapatite & fluorapatite create on exposed organic matrix. The depth of the lesion reduces as the treated lesion gains harder and denser minerals. After being released by re-acidification, silver, fluoride ions penetrate 50-200 microns into dentin and around 25 microns into enamel. The thickness of silver diamine fluoride arrested lesions is 150 microns. Fluoride promotes remineralization, and silver has antimicrobial capabilities. SDF has been coined the "silver-fluoride bullet" for its rapid activity [14]. Artificial lesions that receive silver diamine fluoride remain resistant to biofilm growth and subsequent cavity formation, perhaps because of the residual ionic silver. Since demineralized dentin contains higher concentrations of fluoride and silver, treated dentin that has been demineralized is more susceptible to bacteria that cause caries than treated dentin that has not been demineralized. A "zombie effect" occurs when bacteria that have been killed by silver ions are combined with living bacteria, reactivating the silver and killing the living bacteria [15]. In order to simulate dentine impacted by caries, an artificial lesion was made on dentinal surface of primary molars in current study's-controlled laboratory environment. It should be emphasized that the microstructure of naturally demineralized dentinal surfaces is far more intricate than that of artificially generated carious lesions. There might also be variations in permeability between naturally caries-affected dentine and chemically demineralized dentine since naturally caries-affected dentine contains mineral crystals. This has been assumed to be a significant contributing a component to the dentinal tubules' diminished fluid flow [8]. In accordance to a study by Marquezan *et al.* (2009) [16] artificial approach of caries induction allows for improved standardization and clarity of testing of the specimens because of the small dimensions and close intimacy of caries afflicted primary dentin specimens, as well as the difficulties in acquiring them. Therefore, to replicate carious dentin, demineralized dentin specimens were used in the current study. A one-minute dwell period accompanies SDF application, corresponding to the AAPD 2018 chairside guidance. Here in our study, we applied SDF according to the chairside guidance of the AAPD, providing it a dwell time of one minute and then adding KI for an additional minute until a creamy mix was formed [5].

As the most widely utilized concentration, 38% SDF was used in the current investigation. Commercially, it comes in percentages of 12%, 30%, and 38%. Mei *et al.* [17] explored the effects of various SDF concentrations on matrix metalloproteinases (MMP) and discovered that 38% of SDF had a greater inhibitory effect on MMP than did 30% and 12%. Gao *et al.* [18] extensive review of clinical data suggests that increasing the concentration of SDF from 12% to 38% and changing the frequency of treatment from year to half-yearly will improve its efficacy in caries arrest. Whether in non-carious as well as carious dentin, the black staining may potentially provide a cosmetic problem. When a solution that was saturated of KI and SDF collided, a milky white precipitate containing silver iodide particles was created on the tooth surface. Therefore, a free silver ion with SDF can no longer cause black deposits to form on the teeth, discoloration is avoided [4].

Only competent primary molars devoid of developmental abnormalities or restorations were utilized in the study in order to standardize the results. As a consequence, the teeth were kept for a maximum of one month in a 0.1% thymol solution, which also possesses antibacterial qualities. The shear bond strength of the dentin was not significantly impacted by the teeth being stored in thymol solution for six months, as reported in the Humel *et al.* [19] experiment. Tosun *et al.* [20] and Titley *et al.* [21], however, found that maintaining teeth in thymol for two months greatly decreased their shear bond strength. Berdan *et al.* [22] claim that teeth sustain their microhardness when exposed to a 0.1% concentration thymol solution. In accordance with a study through Lutgen *et al.* [23], unwashed samples after applying SDF had considerably lower bond strengths than groups that had the SDF precipitate wiped off. As a result, a similar procedure was employed in the current investigation, wherein the teeth's surfaces were cleaned with a distilled water solution for 30 seconds following pre-treatment with SDF + KI.

This study assessed the shear bond strength because clinical effectiveness depends on restorative materials' ability to cling securely to the dentinal surface and withstand the various dislodging forces operating within the mouth cavity. The compressive, tensile, and shear strengths of these forces are used for determining them. The tendency of restorative material to resist forces that may otherwise improve it away from the tooth's framework is known as shear bond strength. It has substantial clinical relevance for the restorative material because of the shearing effect of the major dislodging pressures at the tooth restoration contact. Therefore, better bonding amongst the material and the tooth will be achieved by stronger shear bonding [24]. The outcomes revealed a noteworthy distinction between experimental & control groups. Group 3 samples (1.93 MPa) that undergone preliminary treatment with SDF+KI before being restored with type IX GIC had the greatest mean SBS, followed by group 1 samples (1.49 MPa) that underwent direct restoration of the demineralized dentin. The final results of this study's GIC-treated samples are analogous to those of earlier research by Elizabeth Ng *et al.*[9], Gupta *et al.* [25], and Wang *et al.* [26], which demonstrated that applying SDF substantially enhanced bond strength of GIC.

Condensable, packable and highly viscous GICs are the terms used to describe Type IX GICs. Compared to traditional GICs, these have stronger strength, greater wear opposition; and flexural strength because of their smaller particles of glass and higher Powder: Liquid ratio.Type IX GIC is less prevalent to moisture and more resistant to disintegration. The gold standard for ART restorations, according to the World Health Organization, is high viscosity GIC FUJI IX [24]. In the current study, when SDF is applied prior to GIC restoration it helps to deposition of silver and silver oxide on surface of the demineralized dentin leads to improved hardness of demineralized dentin as SDF can increase micro-hardness by stopping the demineralizing reaction, and the fluoride contains helps to initiate the remineralization process. 26 This possibly may have played an important role in the higher bond strength between glass ionomer cement & demineralized dentin to enhance micromechanical interlocking. This might have been the cause that received SDF and KI pre-treatment before having GIC bonding, since they had greater mean shear bond strength.

According to Soeno *et al.* [27] results the reaction intermediates CaF2, AgNO3 and AgPO4 found in SDF would react with protein and hydroxyapatite to limit monomer penetration into the dentin. In comparison to hydroxyapatite crystals, fluorapatite crystals that are produced by SDF fluoride are larger with lesser voids and greater micro-hardness. The hyper-saturated fluoride surface exhibits acid resistance, which could potentially reduce the ion exchange during the acid-base reaction. As a result, the SDF-treated samples' binding strength can be impacted. Thermocycling is a technique used to simulate aging in order to assess the impact of thermal stressors on restorative materials. Consequently, this allowed for the assessment of the many processes taking place in the oral cavity and their relationship to the restorative material's gradual bonding to the dentin. Therefore, 500 thermocycling cycles were performed in this study to simulate two weeks' worth of stress [28]. In the current research, the demineralized tooth samples were not taken from the same tooth; there may have been some variability in the results which is typical of bond strength studies. Additionally, the cement for the glass ionomer cement buildup was mixed by hand, which could lead to some variability and perhaps limit the study. Even if the *in vitro* results are definitive, more investigation is needed to see whether the clinical conditions sustain the effectiveness of the bond strength calculated in current study.

## Conclusion:

More parents are becoming aware of the negative consequences of ECC. The most effective, painless and less intrusive strategy for treating it at this time is SDF. The staining of SDF can be prevented by applying KI and using an aesthetically pleasing substance for restoration. Group 3 Silver diamine fluoride with potassium iodide followed by glass ionomer cement application recorded a higher mean SBS.

## Figures and Tables

**Table 1:** Distribution of Shear Bond Strength According to Group

**Group**	**N**	**MEAN**	**SD**	**f-value**	**p-value**
Type IX GIC	20	1.49	0.38	13.99	<0.0001(s)
(Group 1)					
Type IX GIC + SDF	20	1.09	0.32		
(Group 2)					
Type IX GIC + SDF + KI	20	1.93	0.34		
(Group 3)					
Total	60	1.5	0.48		

**Table 2:** Intergroup comparison of Shear bond strength between variable

		**95% Confidence Interval**				
Group (I)	Group (J)	Mean difference (I-J)	Std. Error	P value	Lower Bound	Upper Bound
Type IX GIC (Group 1)	Type IX GIC + SDF	0.40 (s)	0.15			
	(Group 2)			0.046 (s)	0.0067	0.79
	Type IX GIC + SDF + KI	-0.43(s)	0.15			
	(Group 3)			0.026 (s)	-0.83	-0.04
Type IX GIC + SDF	Type IX GIC + SDF + KI	-0.83 (s)	0.15			
(Group 2)	(Group 3)			<0.0001 (s)	-1.23	-0.44
